# Supplementary material for: p53 Forms Redox-Dependent Protein–Protein Interactions through Cysteine 277
Source: Antioxidants (Basel). 2021 Oct 6;10(10):1578. doi: 10.3390/antiox10101578 (PMC8533633; doi:10.3390/antiox10101578)
Supplement: Supplementary file 1 [file antioxidants-10-01578-s001.zip › antioxidants-1350419-supplementary.pdf]

Supplementary Figures for the article:

p53 Forms Redox-Dependent Protein–Protein Interactions through Cysteine 277

Tao Shi 1, Paulien E. Polderman 1, Marc Pagès-Gallego 1, Robert M. van Es 1, Harmjan R. Vos 1, Boudewijn M. T. Burgering 1,2 and Tobias B. Dansen 1,*

1 Center for Molecular Medicine, Molecular Cancer Research, University Medical Center Utrecht,
Universiteitsweg 100, 3584 CG Utrecht, The Netherlands; t.shi@umcutrecht.nl (T.S.); p.e.polderman@umcutrecht.nl (P.E.P.); m.pagesgallego@umcutrecht.nl (M.P.-G.); r.m.vanes-4@umcutrecht.nl (R.M.v.E.); h.r.vos-3@umcutrecht.nl (H.R.V.); b.m.t.burgering@umcutrecht.nl (B.M.T.B.)
2 Oncode Institute, University Medical Center Utrecht, Universiteitsweg 100, 3584 CG Utrecht, The Netherlands
* Correspondence: t.b.dansen@umcutrecht.nl

Legends to figures

Supplementary Figure S1. reversible redox-dependent protein complexes of p53 are observed upon continuous H2O2 production or under low glucose conditions.

(A) HEK293T cells transiently expressing Flag-p53 (WT) were treated with diamide for 15 min, followed by refreshing the medium. Protein samples were collected at the indicated recovery times (*) after diamide removal.
(B) p53 does not form detectable redox-dependent protein-protein interactions upon prolonged H_2O_2 treatment in HEK293T cells cultured in high glucose media (4.5 g/l).
(C) p53 does not form detectable redox-dependent protein-protein interactions upon prolonged H_2O_2 treatment in H1299 cells cultured in high glucose media (4.5 g/L).
(D) Continuous exogenous H_2O_2 production upon addition of Glucose Oxidase (GOX) induces formation of several p53 redox-dependent protein complexes (indicated by asterisks) in cells cultured in high-glucose (4.5g/L) media. HEK293T cells transiently expressing Flag-p53 (WT) were cultured in high-glucose media or refreshed to PBS 1h prior to treatment with diamide (200 μM, 15min) or H_2O_2 (200 μM, 15min). 100 mU/mL or 1000 mU/mL of GOX was added to cells for the indicated times.
(E) H_2O_2 treatment induces similar p53 redox-dependent complexes as diamide does in cells cultured in low-glucose (1g/L) media. HEK293T cells transiently expressing Flag-p53 (WT) were cultured in low-glucose media or refreshed to PBS 1h prior to the treatment of diamide (200 μM, 15min) or H_2O_2 (200 μM, 15min). 100 mU/mL or 1000 mU/mL of GOX was added to cells for the indicated times. Note that in high glucose media GOX seems to induce more p53 redox-dependent complexes, probably because the enzyme produces more H_2O_2 at this higher substrate concentration.

Supplementary Figure S2. p53 cysteine conservation in vertebrate species.

Alignment of human p53 protein sequences from 5 groups of vertebrate species including fish, amphibians, birds, reptiles and mammals. All protein sequences were downloaded from the ENSEMBL database and the sequence alignment was performed in Jalview (version 15.0) software. The alignments were colored based on the extent of conservation (Threshold 10). Cysteines were further colored in orange in Adobe Illustrator.

Supplementary Figure S3. C277 is required for p53 disulfide-dependent protein interactions.

(A) Evaluation of transcriptional activity of p53 cysteine mutants (to Alanine or Serine) in H1299 cells.
(A) Evaluation of disulfide-dependent protein complexes of p53 cysteine mutants (to Alanine or Serine) in H1299 cells.
(B) Evaluation of disulfide-dependent protein complexes of p53 WT, C182S, C277S and C176S mutants in HEK293T cells.

Supplementary Figure S4. p53 does not form disulfide-dependent homodimers upon diamide treatment.

(A) Flag-p53 and HA-p53 were expressed in HEK293T cells either separately or co-expressed. Immunoprecipitation using anti-Flag or anti-HA was conducted after diamide treatment and Western blots were probed with different fluorescence-conjugated secondary antibodies against Flag (Green) or HA(Red). Flag-p53 (green) and HA-p53(red) were both oxidized to form a complex (*, around 100kDa) with another protein, but not as a homodimer or homo-oligomer as no overlapping signal of green and red was observed. p53 is known to function as a (non-disulfide dependent) tetramer, and Flag-p53 can indeed pull-down HA-p53. The high-molecular weight band containing HA-p53, which is independent of homodimerization of p53 as indicated in the model in (B).

(B) HA-p53 and Flag-p53 form non-covalent tetramers of different composition (other combinations are possible). Diamide treatment induces the formation of disulfide-dependent complexes with an unknown protein. Although Flag-p53 binds HA-p53 non-covalently, HA-p53 can be found to migrate as a redox-sensitive complex in the Flag-directed IP.

Supplementary Figure S5. Reproducible results among 4 replicates in each condition.

(A) WB detection of p53 disulfide-dependent dimerization for the four replicates prepared for MS/MS analysis for each condition.

(B) Bar graph showing the amounts of protein identified by MS in each replicate (R1, R2, R3, R4) and condition.

(C) Box plot showing the distribution of Label-Free quantification (LFQ) intensities of proteins in each replicate and condition. Flag-p53 is indicated as the top outlier with the highest and roughly equal LFQ value in all samples.

(D) Principle component analysis (PCA) of LFQ value in all samples.

Supplementary Figure S6. PANTHER Gene Ontology (GO) analysis of redox dependent-p53
binding partners.

GO enrichment analysis for 167 diamide-induced p53 interactors was performed using the PANTHER Classification System online (http://pantherdb.org/). The top significant terms (p-value < 0.001) in biological process, molecular function and cellular component were presented in the bar plots.

(A) GO analysis of biological process.

(B) GO analysis of molecular function.

(C) GO analysis of cellular component.

Supplementary Figure S7. Validation of the disulfide-dependent interaction between p53 and
14-3-3θ.

HEK293T cells were transfected with Flag-p53 WT in combination with siRNA for 14-3-3θ or a scrambled Ctrl siRNA. After 48h of transfection, IP and WB using a Flag antibody were performed to examine whether the shifted band recognized by the 14-3-3θ antibody is indeed the presumed p53-S-S-14-3-3θ interaction. In the upper left panel, the disulfide-dependent, covalent p53-S-S-14-3-3θ complex (*) is clearly present in the control condition using both Flag (-p53) and 14-3-3θ staining, whereas it is largely gone upon 14-3-3θ depletion (si14-3-3θ) (*). The pattern of other intermolecular disulfide-dependent complexes containing Flag-p53 seems not to be affected by 14-3-3θ knockdown. In the lower right panel, consistently, reduced 14-3-3θ (#) is only observed in

the control condition. A lower level of 14-3-3θ is detected upon siRNA-mediated knock-down in the cleared lysate as well (input).

Supplementary Figure S8. Further Validation of the disulfide-dependent interaction between p53 and 53BP1.

HEK293T cells were transfected with HA-p53 WT or C277S alone or along with GFP-53BP. 48 h after transfection and following 15-min diamide treatment, the p53-53BP1 complex was precipitated using HA-antibody coated beads. The beads were washed with a wash buffer containing low salt (L, 150 mM NaCl), high salt (H, 1M NaCl) or high salt plus 10 mM DTT (D). The wash buffer was otherwise identical to the lysis buffer (see Methods section), minus the alkylating agent. The p53 - 53BP1 interaction was examined by WB using HA and GFP antibodies.

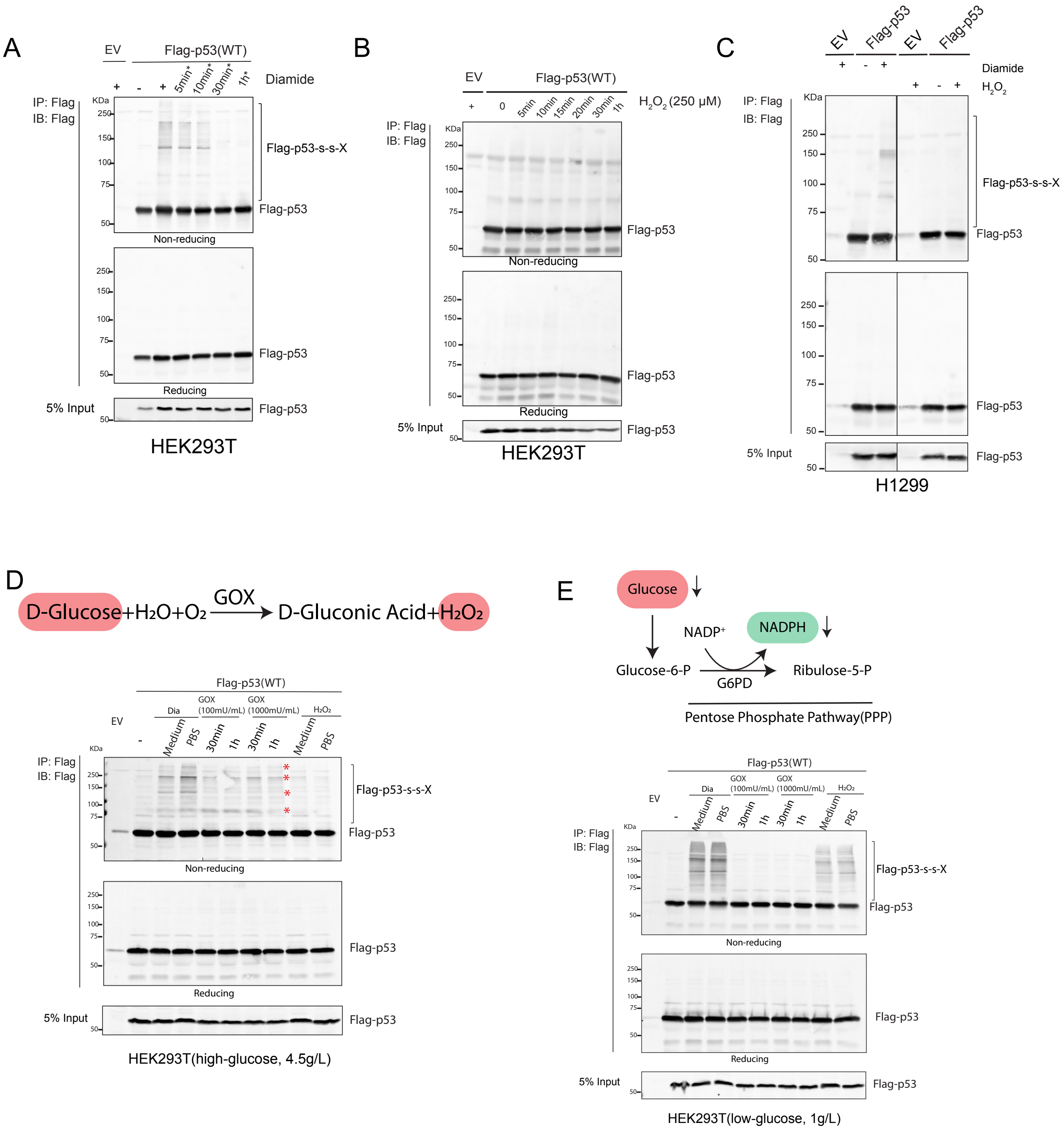
A
EV
Flag-p53(WT)
Diamide
5min*
10min*
30min*
1h*
IP: Flag
IB: Flag
KDa
Flag-p53-s-s-X
Flag-p53
Non-reducing
Reducing
5% Input
HEK293T
B
EV
Flag-p53(WT)
H2O2 (250 μM)
0
5min
10min
15min
20min
30min
1h
Non-reducing
Reducing
5% Input
HEK293T
C
EV
Flag-p53
Diamide
H2O2
H1299
D
D-Glucose+H2O+O2
GOX
D-Gluconic Acid+H2O2
Dia
GOX (100mU/mL)
GOX (1000mU/mL)
Medium
PBS
30min
1h
HEK293T(high-glucose, 4.5g/L)
E
Glucose
NADP+
NADPH
Glucose-6-P
G6PD
Ribulose-5-P
Pentose Phosphate Pathway(PPP)
HEK293T(low-glucose, 1g/L)

Figure S1.

A

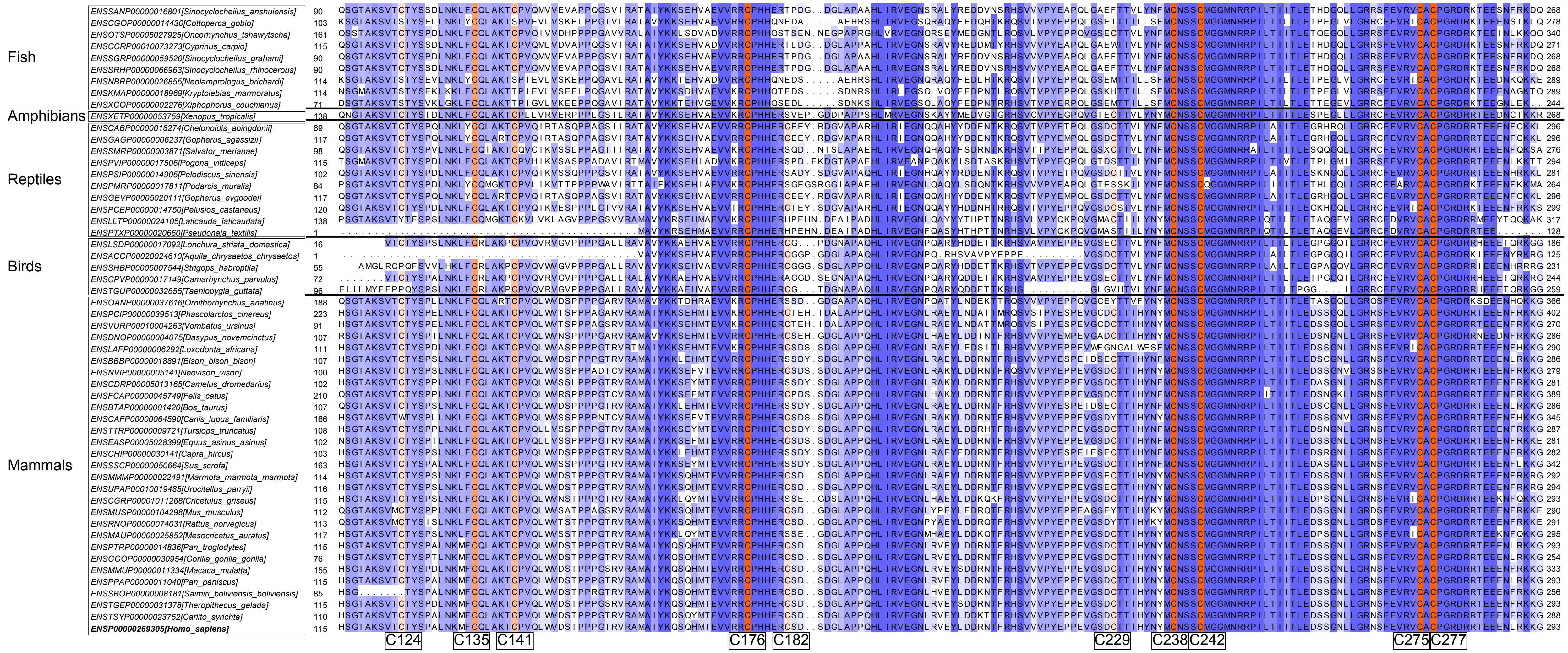
Fish
Amphibians
Reptiles
Birds
Mammals
ENSP00000269305[Homo_sapiens]
C124
C135
C141
C176
C182
C229
C238
C242
C275
C277

Figure S2.

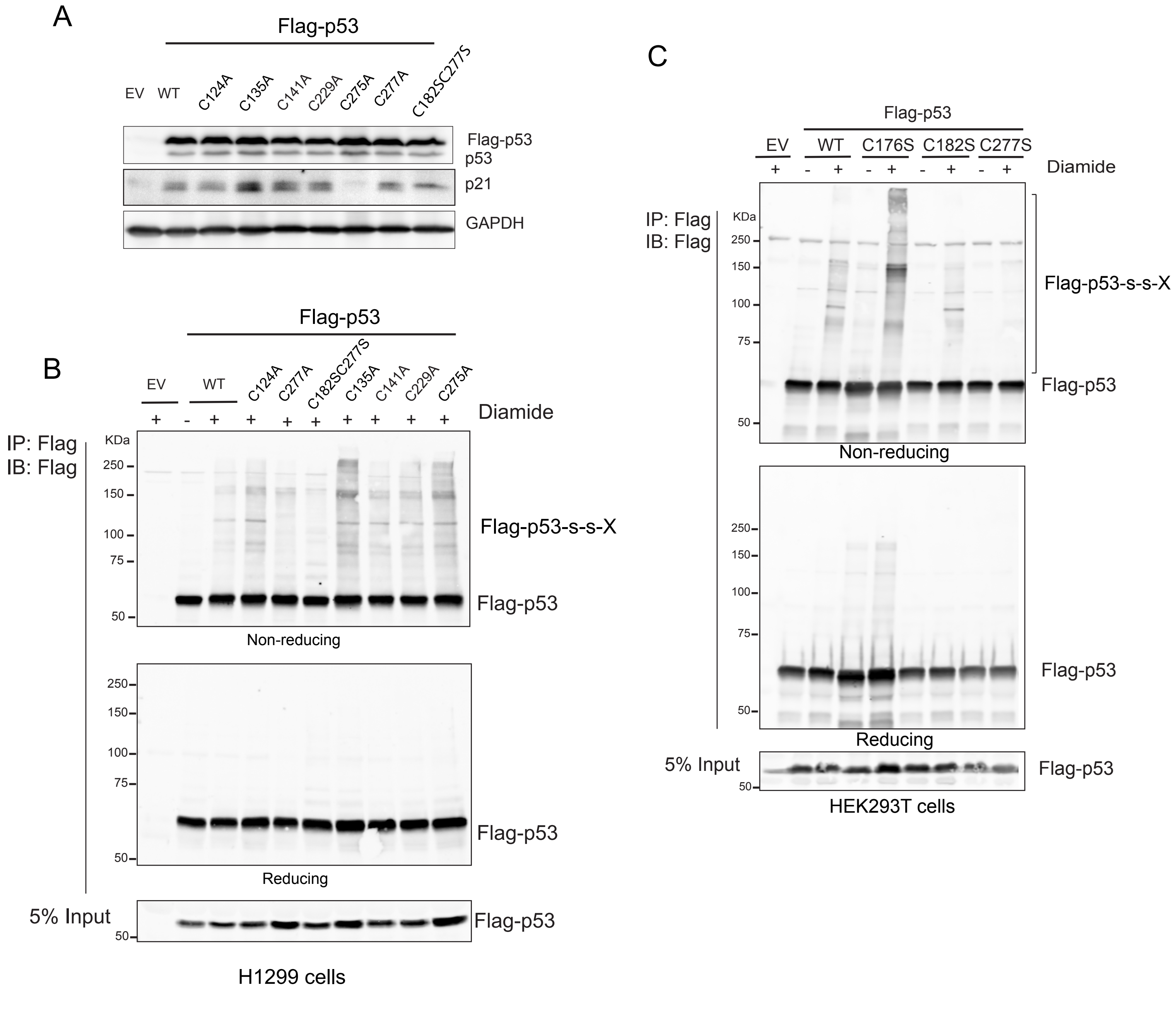

A
Flag-p53
EV
WT
C124A
C135A
C141A
C229A
C275A
C277A
C182SC277S
Flag-p53
p53
p21
GAPDH
B
Flag-p53
EV
WT
C124A
C277A
C182SC277S
C135A
C141A
C229A
C275A
Diamide
IP: Flag
IB: Flag
KDa
250
150
100
75
50
Flag-p53-s-s-X
Flag-p53
Non-reducing
Reducing
5% Input
H1299 cells
C
Flag-p53
EV
WT
C176S
C182S
C277S
Diamide
Flag-p53-s-s-X
Non-reducing
Reducing
5% Input
HEK293T cells

Figure S3.

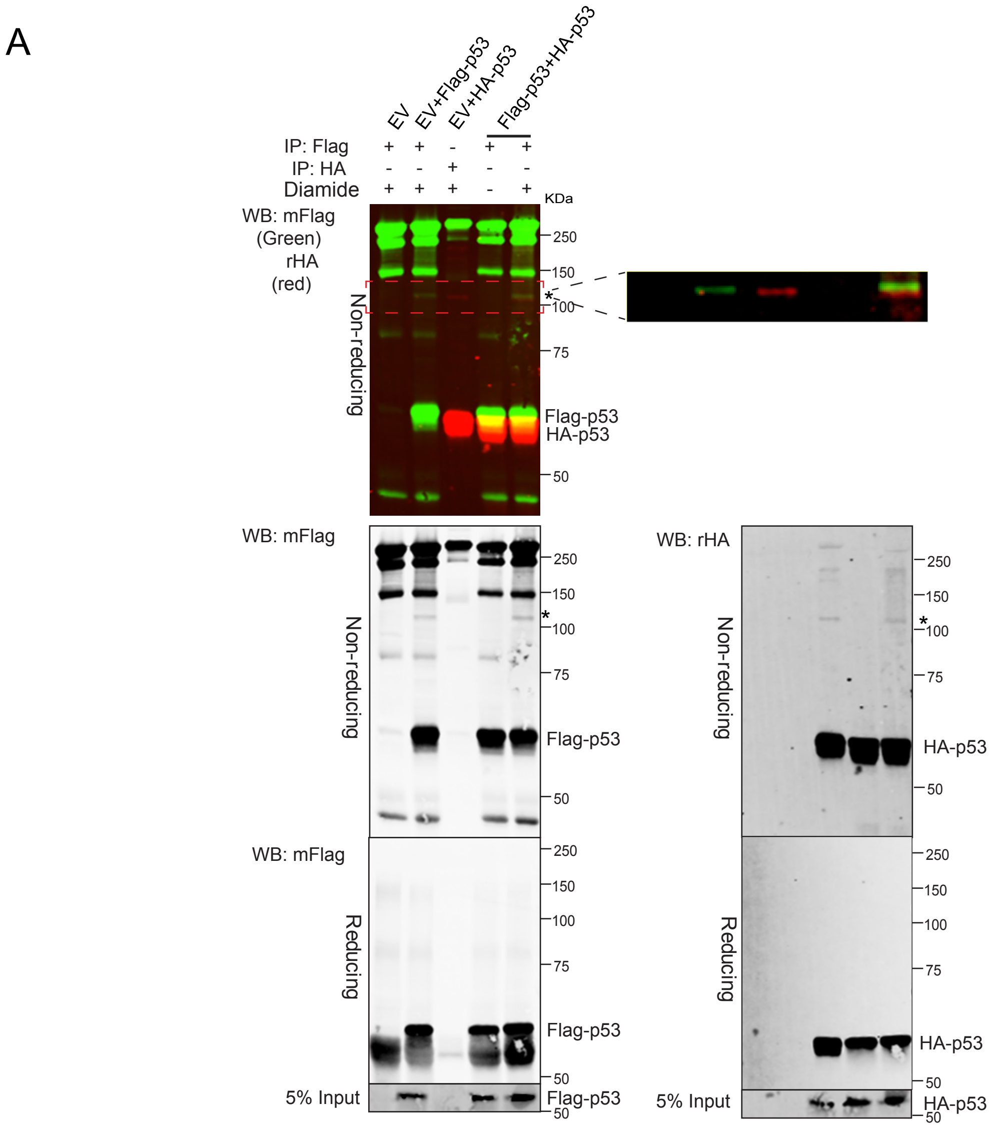
A
EV
EV+Flag-p53
EV+HA-p53
Flag-p53+HA-p53
IP: Flag
IP: HA
Diamide
WB: mFlag
(Green)
rHA
(red)
KDa
250
150
100
75
50
Non-reducing
Flag-p53
HA-p53
WB: mFlag
WB: rHA
Reducing
5% Input

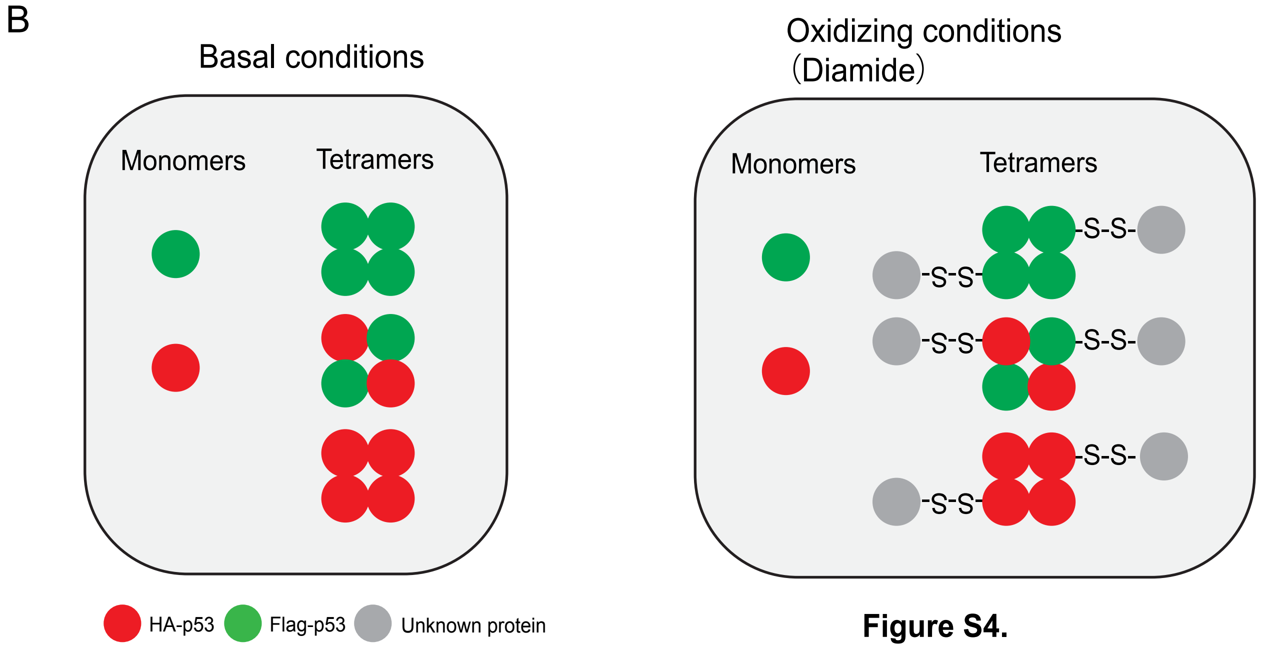
B
Basal conditions
Monomers
Tetramers
Oxidizing conditions
(Diamide)
-S-S-
HA-p53
Flag-p53
Unknown protein

Figure S4.

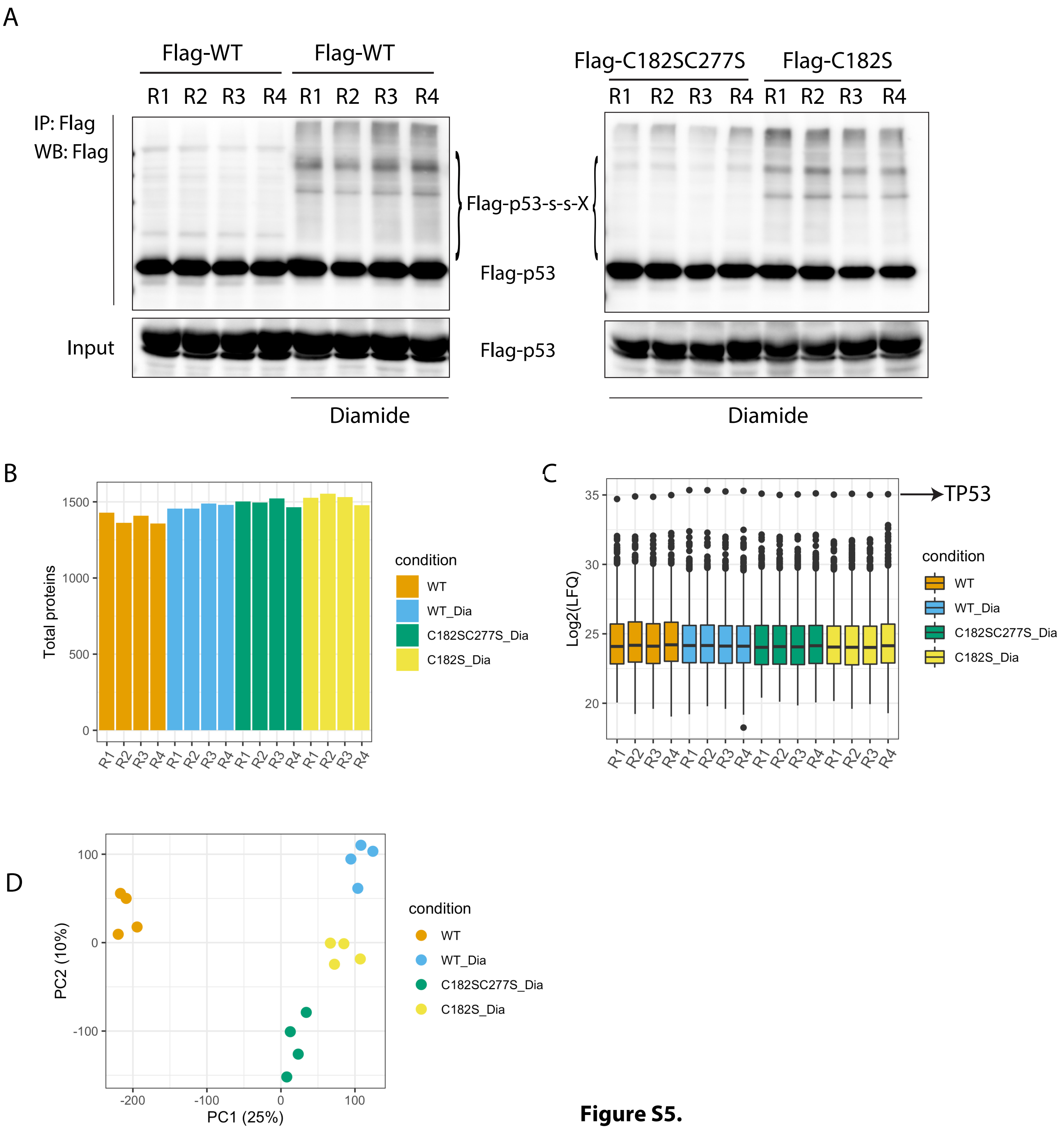
A
Flag-WT
Flag-WT
R1 R2 R3 R4 R1 R2 R3 R4
IP: Flag
WB: Flag
Flag-p53-s-s-X
Flag-p53
Input
Flag-p53
Diamide
Flag-C182SC277S
Flag-C182S
R1 R2 R3 R4 R1 R2 R3 R4
Diamide
B
Total proteins
1500
1000
500
0
R1 R2 R3 R4 R1 R2 R3 R4 R1 R2 R3 R4 R1 R2 R3 R4
condition
WT
WT_Dia
C182SC277S_Dia
C182S_Dia
C
Log2(LFQ)
35
30
25
20
TP53
R1 R2 R3 R4 R1 R2 R3 R4 R1 R2 R3 R4 R1 R2 R3 R4
condition
WT
WT_Dia
C182SC277S_Dia
C182S_Dia
D
PC2 (10%)
100
0
-100
PC1 (25%)
-200
-100
0
100
condition
WT
WT_Dia
C182SC277S_Dia
C182S_Dia

Figure S5.

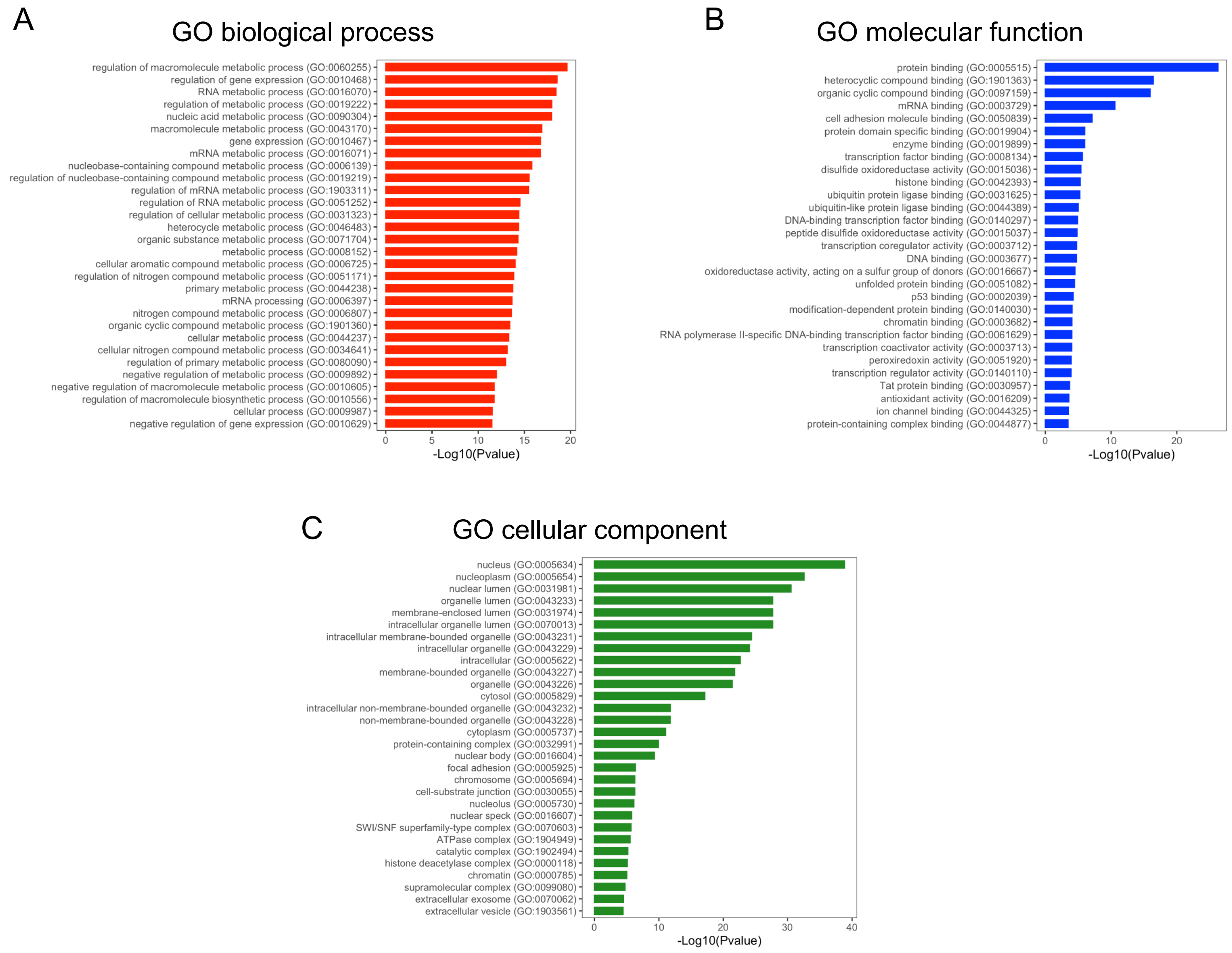

A
GO biological process
regulation of macromolecule metabolic process (GO:0060255)
regulation of gene expression (GO:0010468)
RNA metabolic process (GO:0016070)
regulation of metabolic process (GO:0019222)
nucleic acid metabolic process (GO:0090304)
macromolecule metabolic process (GO:0043170)
gene expression (GO:0010467)
mRNA metabolic process (GO:0016071)
nucleobase-containing compound metabolic process (GO:0006139)
regulation of nucleobase-containing compound metabolic process (GO:0019219)
regulation of mRNA metabolic process (GO:1903311)
regulation of RNA metabolic process (GO:0051252)
regulation of cellular metabolic process (GO:0031323)
heterocycle metabolic process (GO:0046483)
organic substance metabolic process (GO:0071704)
metabolic process (GO:0008152)
cellular aromatic compound metabolic process (GO:0006725)
regulation of nitrogen compound metabolic process (GO:0051171)
primary metabolic process (GO:0044238)
mRNA processing (GO:0006397)
nitrogen compound metabolic process (GO:0006807)
organic cyclic compound metabolic process (GO:1901360)
cellular metabolic process (GO:0044237)
cellular nitrogen compound metabolic process (GO:0034641)
regulation of primary metabolic process (GO:0080090)
negative regulation of metabolic process (GO:0009892)
negative regulation of macromolecule metabolic process (GO:0010605)
regulation of macromolecule biosynthetic process (GO:0010556)
cellular process (GO:0009987)
negative regulation of gene expression (GO:0010629)
0
5
10
15
20
-Log10(Pvalue)
B
GO molecular function
protein binding (GO:0005515)
heterocyclic compound binding (GO:1901363)
organic cyclic compound binding (GO:0097159)
mRNA binding (GO:0003729)
cell adhesion molecule binding (GO:0050839)
protein domain specific binding (GO:0019904)
enzyme binding (GO:0019899)
transcription factor binding (GO:0008134)
disulfide oxidoreductase activity (GO:0015036)
histone binding (GO:0042393)
ubiquitin protein ligase binding (GO:0031625)
ubiquitin-like protein ligase binding (GO:0044389)
DNA-binding transcription factor binding (GO:0140297)
peptide disulfide oxidoreductase activity (GO:0015037)
transcription coregulator activity (GO:0003712)
DNA binding (GO:0003677)
oxidoreductase activity, acting on a sulfur group of donors (GO:0016667)
unfolded protein binding (GO:0051082)
p53 binding (GO:0002039)
modification-dependent protein binding (GO:0140030)
chromatin binding (GO:0003682)
RNA polymerase II-specific DNA-binding transcription factor binding (GO:0061629)
transcription coactivator activity (GO:0003713)
peroxiredoxin activity (GO:0051920)
transcription regulator activity (GO:0140110)
Tat protein binding (GO:0030957)
antioxidant activity (GO:0016209)
ion channel binding (GO:0044325)
protein-containing complex binding (GO:0044877)
0
10
20
-Log10(Pvalue)
C
GO cellular component
nucleus (GO:0005634)
nucleoplasm (GO:0005654)
nuclear lumen (GO:0031981)
organelle lumen (GO:0043233)
membrane-enclosed lumen (GO:0031974)
intracellular organelle lumen (GO:0070013)
intracellular membrane-bounded organelle (GO:0043231)
intracellular organelle (GO:0043229)
intracellular (GO:0005622)
membrane-bounded organelle (GO:0043227)
organelle (GO:0043226)
cytosol (GO:0005829)
intracellular non-membrane-bounded organelle (GO:0043232)
non-membrane-bounded organelle (GO:0043228)
cytoplasm (GO:0005737)
protein-containing complex (GO:0032991)
nuclear body (GO:0016604)
focal adhesion (GO:0005925)
chromosome (GO:0005694)
cell-substrate junction (GO:0030055)
nucleolus (GO:0005730)
nuclear speck (GO:0016607)
SWI/SNF superfamily-type complex (GO:0070603)
ATPase complex (GO:1904949)
catalytic complex (GO:1902494)
histone deacetylase complex (GO:0000118)
chromatin (GO:0000785)
supramolecular complex (GO:0099080)
extracellular exosome (GO:0070062)
extracellular vesicle (GO:1903561)
0
10
20
30
40
-Log10(Pvalue)

Figure S6.

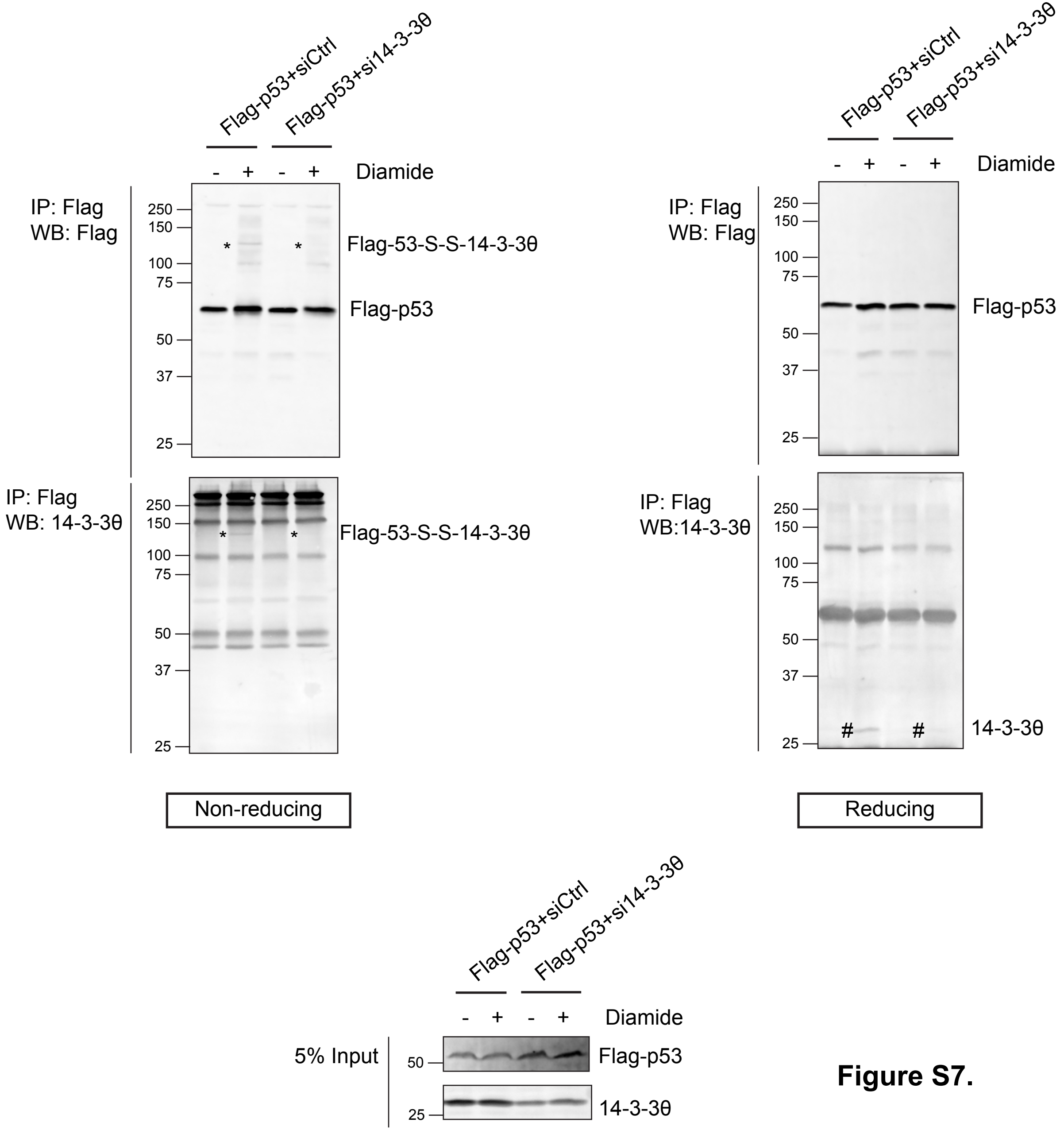

Flag-p53+siCtrl
Flag-p53+si14-3-3θ
- + - + Diamide
IP: Flag
WB: Flag
250
150
100
75
50
37
25
*
*
Flag-53-S-S-14-3-3θ
Flag-p53
IP: Flag
WB: 14-3-3θ
Flag-53-S-S-14-3-3θ
Non-reducing
IP: Flag
WB:14-3-3θ
#
#
14-3-3θ
Reducing
5% Input
Flag-p53
14-3-3θ

Figure S7.

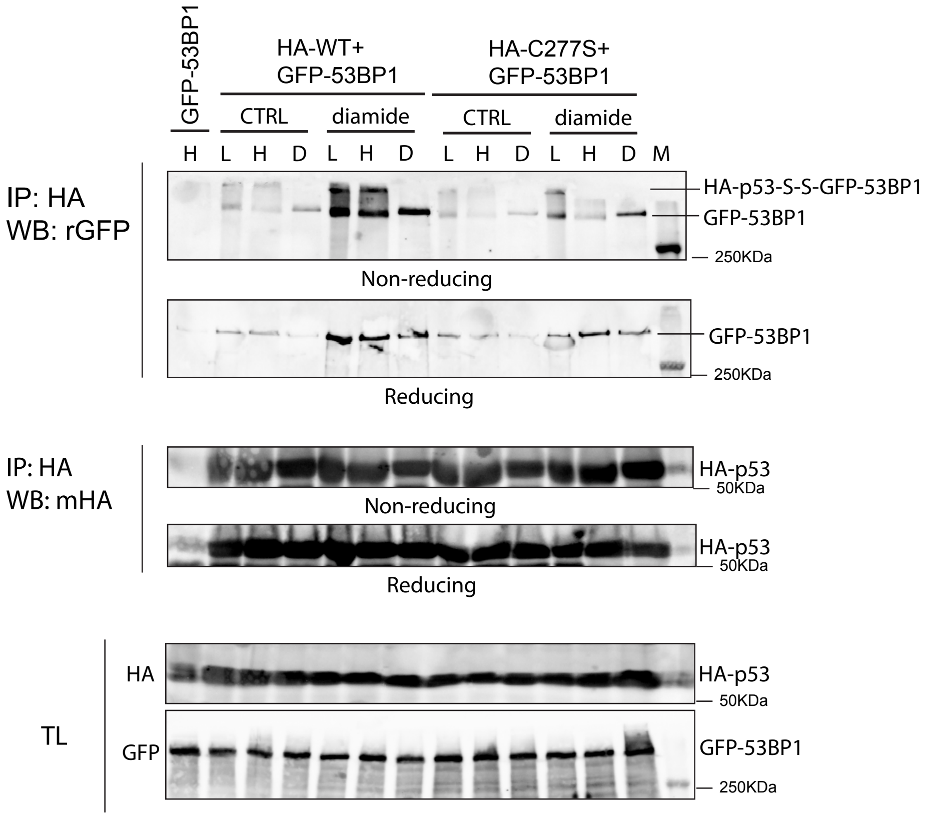
GFP-53BP1
HA-WT+
GFP-53BP1
HA-C277S+
GFP-53BP1
CTRL
diamide
CTRL
diamide
H L H D L H D L H D L H D M
IP: HA
WB: rGFP
HA-p53-S-S-GFP-53BP1
GFP-53BP1
250KDa
Non-reducing
GFP-53BP1
250KDa
Reducing
IP: HA
WB: mHA
HA-p53
50KDa
Non-reducing
HA-p53
50KDa
Reducing
TL
HA
HA-p53
50KDa
GFP
GFP-53BP1
250KDa

Figure S8.

Table S1. Primers for mutagenesis PCR.

Mutant	Primers(5' to 3')
p53C124S	Forward: GGGACAGCCAAGTCTGTGACTAGCACGTACTCCCCTGCCCTCA<br>Reverse: TGAGGGCAGGGGAGTACGTGCTAGTCACAGACTTGGCTGTCCC
p53C124A	Forward: GGGACAGCCAAGTCTGTGACTGCCACGTACTCCCCTGCCCTCA<br>Reverse: TGAGGGCAGGGGAGTACGTGGCAGTCACAGACTTGGCTGTCCC
p53C135S	Forward: CCTGCCCTCAACAAGATGTTTAGCCAACTGGCCAAGACCTGC<br>Reverse: GCAGGTCTTGGCCAGTTGGCTAAACATCTTGTTGAGGGCAGG
p53C135A	Forward: CCTGCCCTCAACAAGATGTTTGCCCAACTGGCCAAGACCTGC<br>Reverse: GCAGGTCTTGGCCAGTTGGGCAAACATCTTGTTGAGGGCAGG
p53C141S	Forward: GCCAACTGGCCAAGACCAGCCCTGTGCAGCTGTGGGT<br>Reverse: ACCCACAGCTGCACAGGGCTGGTCTTGGCCAGTTGGCA
p53C141A	Forward: TGCCAACTGGCCAAGACCGCCCCTGTGCAGCTGTGGGT<br>Reverse: ACCCACAGCTGCACAGGGGCGGTCTTGGCCAGTTGGCA
p53C176S	Forward: ACGGAGGTTGTGAGGCGCAGCCCCCACCATGAGCGCTG<br>Reverse: CAGCGCTCATGGTGGGGGCTGCGCCTCACAACCTCCGT
p53C182S	Forward: TGCCCCCACCATGAGCGCAGCTCAGATAGCGATGGTCT<br>Reverse: AGACCATCGCTATCTGAGCTGCGCTCATGGTGGGGGCA
p53C182A	Forward: TGCCCCCACCATGAGCGCGCCTCAGATAGCGATGGTCT<br>Reverse: AGACCATCGCTATCTGAGGCGCGCTCATGGTGGGGGCA
p53C229S	Forward: CCTGAGGTTGGCTCTGACAGTACCACCATCCACTACAACT<br>Reverse: AGTTGTAGTGGATGGTGGTACTGTCAGAGCCAACCTCAGG
p53C229A	Forward: CCTGAGGTTGGCTCTGACGCTACCACCATCCACTACAACT<br>Reverse: AGTTGTAGTGGATGGTGGTAGCGTCAGAGCCAACCTCAGG
p53C275S	Forward: ACAGCTTTGAGGTGCGTGTTAGTGCCTGTCCTGGGAGAGAC<br>Reverse: GTCTCTCCCAGGACAGGCACTAACACGCACCTCAAAGCTGT
p53C275A	Forward: ACAGCTTTGAGGTGCGTGTTGCTGCCTGTCCTGGGAGAGAC<br>Reverse: GTCTCTCCCAGGACAGGCAGCAACACGCACCTCAAAGCTGT
p53C277S	Forward: TTGAGGTGCGTGTTTGTGCCAGTCCTGGGAGAGACCGGC<br>Reverse: GCCGGTCTCTCCCAGGACTGGCACAAACACGCACCTCAA
p53C277A	Forward: TGAGGTGCGTGTTTGTGCCGCTCCTGGGAGAGACCGGCGCA<br>Reverse: TGCGCCGGTCTCTCCCAGGAGCGGCACAAACACGCACCTCA

Table S2. Diamide-dependent interactors of p53.

	Diamide-dependent interactors	The interaction with the proteins indicated in Red is significantly enriched in all three comparisons p53 WT vs p53 WT +Diamide, p53 WT +Diamide vs p53 C182SC277S +Diamide and p53 C182S + Diamide vs p53 C182SC277S +Diamide, but not in the comparison p53 WT +Diamide vs p53 C182S +Diamide. The binding of these proteins therefore likely depends on p53 C277.
1	TLK2	
2	ARNT	
3	C18orf25	
4	PPP1R2;PPP1R2P3	
5	UBXN7	
6	RANBP3	
7	COPS2	
8	DCTPP1	
9	TP53BP1	
10	HDGFRP2	
11	PCYT1A	
12	YWHAE	
13	SAFB	
14	MDM2;MDM2 isoform KB9;mdm2	
15	SLC4A1AP	
16	YWHAZ	
17	PSME3	
18	DFFA	
19	YWHAQ	
20	RPRD1A	
21	ZNF428	
22	TTC1	
23	CNN2	
24	ARID3B	
25	PUS7	
26	CTDP1	
27	NCL	
28	RAD18	
29	SRCAP	
30	GLMN	
31	SMAP;C11orf58	
32	ELL	
33	RBM17	
34	NCOR1	
35	RCC2	
36	TRIM33	
37	FTO	
38	BCLAF1	
39	TUBA1A;TUBA3E	
40	PPAN-P2RY11;PPAN	
41	GSTP1	
42	PSMD9	
43	CPSF7	
44	RNF126	
45	GRB2	
46	NR2C2AP	
47	SMARCC1	
48	POGK	
49	CARHSP1	
50	PTBP1	
51	CSTF2	

52	CDKN2A	
53	RNGTT	
54	TOX4	
55	JUN	
56	YWHAB	
57	ZFP36L2;ZFP36L1	
58	SUPT4H1	
59	BPTF	
60	SRSF5	
61	SAFB2	
62	CTTN	
63	ZXDC;ZXDA;ZXDB	
64	CIAPIN1	
65	DUS1L	
66	DNAJC9	
67	PHAX	
68	UBTF	
69	CD2BP2	
70	HMGB1;HMGB1P1	
71	MNAT1	
72	FOXK2	
73	RING1	
74	SRSF6	
75	EP400	
76	GINS3	
77	XPO5	
78	NELFA	
79	PDCD11	
80	VBP1	
81	NME1;NME2;NME1-NME2;NME2P1	
82	EIF4B	
83	ZPR1	
84	CHERP	
85	GLRX3	
86	TXN	
87	PRDX2	
88	YWHAG	
89	CNBP	
90	CNN3	
91	SERBP1	
92	SH3GL1	
93	POLR1E	
94	CACYBP	
95	AASDHPPT	
96	PHF8	
97	IMPDH2	
98	TRIM24	
99	PRDX6	
100	TXLNA	
101	FOXK1	
102	ANP32E	
103	PNN	

104	BOLA2	
105	ACIN1	
106	CHD8	
107	PDLIM1	
108	TXNL1	
109	FLNB	
110	TTC4	
111	SRSF3	
112	PFDN2	
113	NUDC	
114	SNW1	
115	YARS	
116	KHDRBS1	
117	TCOF1	
118	ARMC6	
119	POGZ	
120	USP48	
121	NUDCD2	
122	HIRIP3	
123	MAP4	
124	EIF5A;EIF5A2;EIF5AL1	
125	MBD3	
126	RBM25	
127	IRF2BPL	
128	TPM3;DKFZp686J1372	
129	PPP5C	
130	KIF4A	
131	NASP	
132	UBXN1	
133	MCMBP	
134	CHD1	
135	UBA1	
136	CBX3	
137	RPS12	
138	DNAJA1	
139	TCEA1	
140	HCFC1	
141	AIP	
142	TMPO	
143	STIP1	
144	U2SURP	
145	ZRANB2	
146	USP5	
147	RBM26	
148	IRF2BP1	
149	HDLBP	
150	NPM1	

151	FLNA	
152	PRDX1	
153	DNAJC7	
154	HNRNPA3	
155	TLN1	
156	RNH1	
157	FHL1	
158	KHSRP	
159	SRRM2	
160	HSPH1	
161	ZC3H14	
162	CCT2	
